# Osteosarcoma of the rib

**DOI:** 10.2349/biij.4.1.e7

**Published:** 2008-01-01

**Authors:** WY Lim, S Ahmad Sarji, YI Yik, TM Ramanujam

**Affiliations:** 1 Department of Biomedical Imaging, Faculty of Medicine, University of Malaya, Kuala Lumpur, Malaysia; 2 Department of Surgery, Faculty of Medicine, University of Malaya, Kuala Lumpur, Malaysia

**Keywords:** Osteosarcoma, rib

## Abstract

This case describes the radiological-surgical correlation of a rare case of osteosarcoma of the rib in a 15-year-old boy. Successful repair of his chest wall defect using a wire mesh following extensive surgical resection of the tumour is highlighted, such a procedure being the first instituted at our centre.

## INTRODUCTION

All chest wall tumours in the paediatric population must be assumed to be malignant. The differential diagnoses include Ewings sarcoma, rhabdomyosarcoma, chondrosarcoma, primitive neuroectodermal tumours (PNET) or Askin tumours, other sarcomas and metastatic lesions in the ribs. Osteosarcoma occurs principally in the long bones while Ewings sarcoma is frequently seen in flat bones like the ribs and pelvic bones. Osteosarcoma occurring as a primary tumour in the rib is rare [1, 2]. This paper describes a rare case of osteosarcoma of the rib in a 15-year-old boy, its imaging features and surgical management.

## CASE REPORT

**Figure 1:**
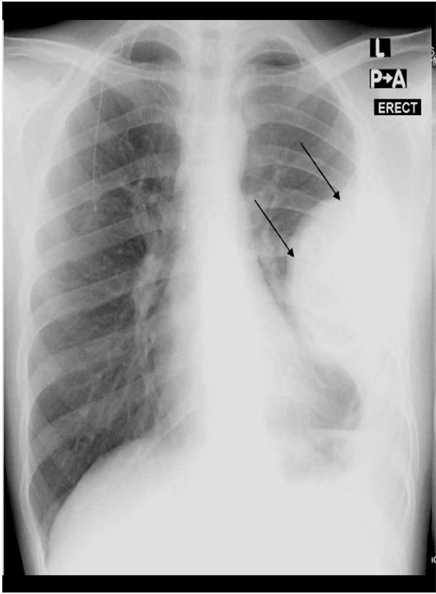
Chest radiograph showing a large pleural-based mass in the left hemithorax (arrows), with underlying rib destruction and a pleural effusion.

**Figure 2:**
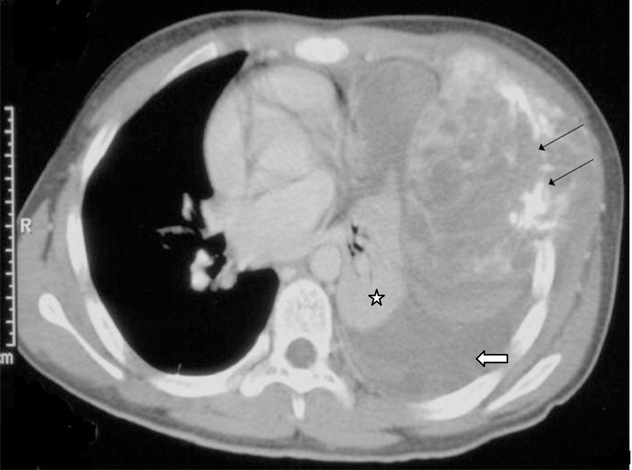
A contrast enhanced CT examination of the chest showing a large heterogenously enhancing solid mass arising from the skeletal chest wall with lytic destruction of the rib and calcifications (arrows). There is a moderate-sized pleural effusion (block arrow) and underlying lung collapse and consolidation (star).

A 15-year-old Chinese boy presented with left-sided chest pain, loss of appetite and weight, and low grade fever for 6 weeks followed by cough and shortness of breath for a week prior to admission. On examination, he was pale and febrile with a temperature of 38.5ºC. Examination of his chest revealed reduction of chest movement on the left side with reduced air entry into the left lung. There were no masses palpable. Haematological investigation revealed haemoglobin of 8.0 g/dL and a normal white cell count. Other blood investigations were unremarkable. A chest radiograph showed a large pleural mass in the left hemithorax, with rib destruction and a pleural effusion but no significant shift of midline structures (Figure 1). A CT examination of the chest showed a large heterogenously enhancing mass arising from left chest wall with lytic destruction of the fourth rib and coarse calcifications. There was a left pleural effusion with underlying lung collapse consolidation. There were no lung nodules in the right lung to suggest metastases (Figure 2). Due to the presence of calcifications in the tumour, a provisional diagnosis of osteogenic sarcoma with a differential diagnosis of chondrosarcoma was made. Pleural aspirate yielded bloody exudate. Cytology was negative and culture of the aspirate showed no growth. A tru-cut biopsy of the mass revealed osteogenic sarcoma. Histopathology findings showed a cellular tumour composed of diffuse sheets of cells with pleomorphic nuclei and basophilic cytoplasm with presence of multinucleated cells with mitotic figures. There were also areas of calcification and osteoid formation. This was in keeping with a tumour originating from the bone.

**Figure 3:**
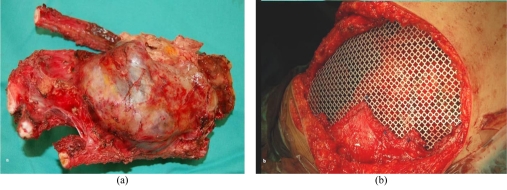
(a) Gross appearance of the resected chest wall and the tumour (b) Appearance of the surgical site after resection of the tumour showing the titanium mesh covering the chest wall defect.

**Figure 4:**
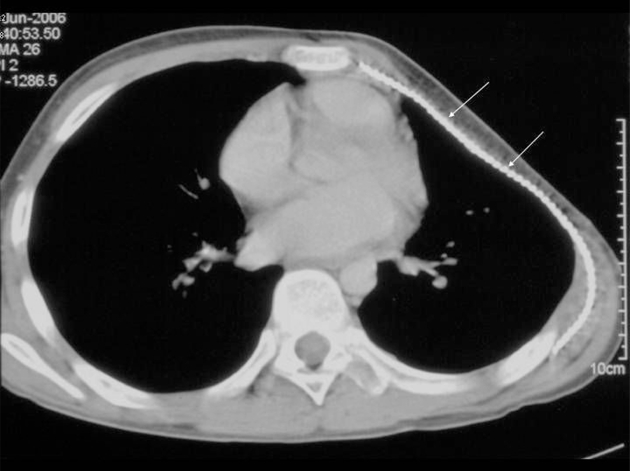
CT examination of the chest post-chemotherapy and chest wall surgery showing deformity of the left chest wall at site of extensive rib resection. The titanium mesh is seen in situ (arrows). There is no evidence of tumour recurrence.

He underwent three cycles of chemotherapy. Post-chemotherapy CT examination for assessment showed reduction of pleural effusion but insignificant improvement in the tumour size. He was commenced on second-line chemotherapy for sarcoma consisting of Ifosfamide and Etoposide and completed two cycles. A CT examination done after completion of this chemotherapy regime again showed no significant reduction in tumour size. A decision was made for surgical excision of the tumour. At surgery, a necrotic tumour mass measuring 9 cm by 13 cm in size arising from the antero-lateral region of the left chest wall and surrounded by thickened pleura was found extended from the third to the seventh intercostal spaces. It was adherent to the left hemidiaphragm and causing significant compression of the left lung. The third to seventh ribs on the left side was resected en bloc followed by total clearance of associated necrotic tissue and diseased pleura. A left thoracoplasty and reconstruction of the affected chest wall defect using a titanium mesh was done (Figure 3). He recovered well from the surgery. There was no further chemotherapy given to the patient after surgery. A CT examination done 3 months later showed deformity of the left chest wall and the titanium mesh in situ (Figure 4). There was no evidence of tumour recurrence, pleural effusion, enlarged mediastinal nodes or focal lung lesions. The patient remained well ten months after surgery.

## DISCUSSION

Osteosarcoma and Ewings sarcoma make up the large majority of pediatric bone tumours. Whilst osteosarcoma occurs principally in the long bones, Ewings sarcoma is frequently seen in flat bones like the ribs and pelvic bones. Osteosarcoma occurring as a primary tumour in the ribs is rare [1, 2] although a few cases of osteosarcoma arising as metastatic involvement in the ribs have been reported [1].

The peak incidence of osteosarcoma is in the second decade of life with another peak occurring in older individuals after radiation or Paget's disease. Burt et al found the majority of chest wall sarcomas were related to previous irradiation to the chest [3]. A few recent cases of osteosarcoma of the rib have been reported in older children ranging from 7 to 13 years of age [1, 2, 4, 5]. The youngest patient with osteosarcoma of the rib reported in the literature to this date is a 7-year-old girl [4]. Our patient is a 15-year-old adolescent. Histology studies showed the majority of the tumours were of the highly malignant type. Extremely well differentiated osteosarcoma has been reported occurring in the rib of a 45-year-old female [6]. It appears that the more malignant type of osteosarcoma occurs in younger children.

The commonest presentation of osteosarcoma of the rib is pain and a palpable chest wall mass. The condition poses a significant diagnostic challenge. The lesion is usually detected on a chest radiograph as a soft tissue mass. Periosteal reaction, rib destruction and calcifications within the mass may be difficult to visualise and assess, given the limitations of a chest radiograph compared to the site of the tumour if it were in a long bone. CT examination is useful for identification and characterisation of the mass. Often, the epicentre of the mass can be localised to the rib. Invasion to deeper structures like the muscles, pleura and lungs can also be accurately assessed. Magnetic resonance imaging (MRI) may not offer any additional information for definitive management as it is a tumour originating from the bone, thus CT would be a more cost effective modality for evaluating the tumour. This was proven true in this case where CT determined poor response to chemotherapy while assessment of involvement of deeper structures allowed the surgeons to plan the operation. In a case of osteosarcoma in a posterior rib with involvement of the lumbar muscles of the back, MRI was used to assess involvement of the spinal canal [2].

Osteosarcoma is derived from primitive bone-forming mesenchymal stem cells and most often occur near the metaphyseal portion of the long bones [7]. The histologic subtypes of osteosarcoma described in the literature include the parosteal, periosteal, well-differentiated intramedullary, fibroblastic, osteoblastic and fibrosarcomatous types [7]. For osteoasarcoma of the rib, a wide surgical excision followed by adjuvant chemotherapy increases the chance of a relapse-free survival of the patient [4]. Surgery should include resection of the full thickness of the chest wall with wide margins that may include the adjacent ribs, intercostal muscles, pleura and vertebrae. Large chest wall defects after resection may require tissue flaps or mesh materials. In a review of surgical treatment of many types of primary chest wall tumours in 41 patients by Athanassiadi K et al., the majority had surgical treatment consisting of wide resection with the use of synthetic mesh in 5 patients [8]. Marlex and metal meshes have been used for this purpose. The disadvantage of the metal mesh material is fragmentation over several months while the Marlex mesh often results in non-contouring of the material, producing compromised ipsilateral pleural space with the possibility of compromised pulmonary function [2]. The surgeons decided to use a titanium mesh, which is usually used in neurosurgical procedures in the skull, because of the good contouring properties of this material. In this case, the titanium mesh was used successfully for the first time in the authors’ institution for a surgery of this kind.

Postoperative multi-agent chemotherapy has improved these patients' survival, reducing the risk of both local and distant relapse. However, there are also reviews which report no apparent difference between the patients treated with surgery alone and those treated with both surgery and chemotherapy. Overall survival despite modern adjuvant chemotherapy is noted to be 27% at 5 years [2]. Osteosarcoma of the flat bones are rarely associated with metastasis in contrast to osteosarcoma at other sites [1]. In this patient, despite the large tumour mass with aggressive features on imaging and poor response to chemotherapy, there was no evidence of metastases.

In summary, this paper presents a rare case of primary osteosarcoma of the rib and emphasise that this condition should be considered in the differential diagnoses of children and adolescents presenting with a chest wall tumour. As metastases at presentation is uncommon, early diagnosis and aggressive surgical management will help to improve outcome. CT remains the essential imaging modality for accurate primary evaluation of the tumour mass, identification of metastatic disease, evaluation of tumour response to chemotherapy and postoperative evaluation of recurrent neoplasm, all of which have important prognostic implications.
